# Severe Chest Pain due to *N*-Acetylcysteine-Induced Esophagitis

**DOI:** 10.1155/2019/8057259

**Published:** 2019-10-20

**Authors:** Weihong Wang, Yu Zhang, Yi Liu, Lei Xu, Dingmei Shi

**Affiliations:** Department of Gastroenterology, Ningbo First Hospital, Ningbo, China

## Abstract

We report an unusual case of severe chest pain caused by *N*-acetylcysteine-induced esophagitis. An 81-year-old Chinese man with a history of interstitial lung disease was admitted to our hospital with intermittent arrhythmia that began 5 days ago. The patient presented with complaints of cough, sputum, and shortness of breath. Cefminox injections and *N*-acetylcysteine tablets were prescribed to improve respiratory symptoms. The patient developed severe chest pain and odynophagia 4 hours after swallowing the *N*-acetylcysteine tablet while in the decubitus position. Upper gastrointestinal endoscopy revealed four discrete areas of ulcerations measuring approximately 1 cm at the midesophageal level. The distance between the foci and the incisors was approximately 24 cm. The patient continued the *N*-acetylcysteine orally, which was administered in powdered form with more water while in the upright position. Pantoprazole and hydrotalcite were also administered to the patient. The symptoms subsided, and a follow-up endoscopy after 20 days showed that the ulcers healed. This case highlights that seemingly safe drugs such as *N-*acetylcysteine can lead to severe chest pain if ingested inappropriately.

## 1. Introduction

*N-*Acetylcysteine (NAC) has been used for the treatment of numerous disorders including cardiac injury and bronchitis. The efficacy and safety have been established for several decades [1, 2]. Several oral drugs have been reported to cause pill-induced esophagitis. However, esophagus injury caused by NAC is rarely reported. We report an unusual case of severe chest pain caused by *N-*acetylcysteine-induced esophagitis.

## 2. Case Report

**Figure 1:**
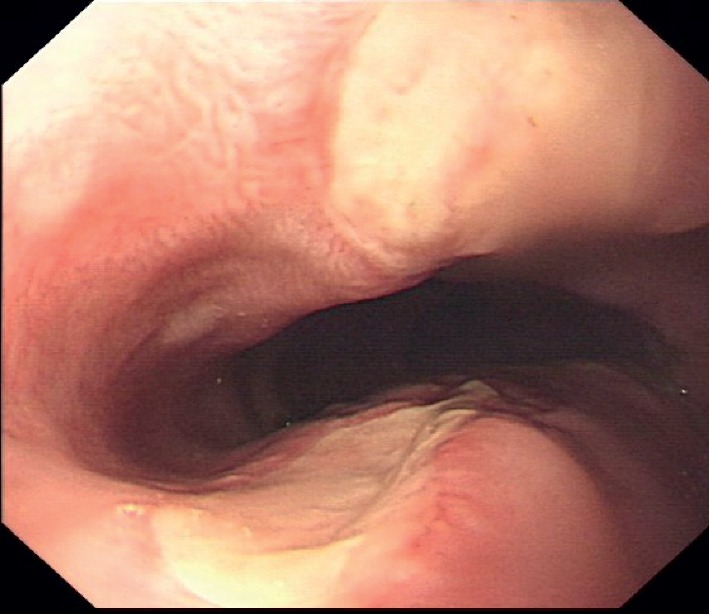
Endoscopic appearance of *N-*acetylcysteine pill-induced esophagitis.

An 81-year-old Chinese man with a history of interstitial lung disease was admitted to the Department of Cardiology at our hospital, and he presented with intermittent arrhythmia that began 5 days ago. The patient presented with complaints of cough, sputum, and shortness of breath. Cefminox injections and *N-*acetylcysteine tablets were prescribed to improve respiratory symptoms. Cefminox was injected twice a day with 1 g dosage for each injection, and 0.6 g *N-*acetylcysteine tablets were administered twice a day. No other tablets or herbal treatments were administered. The patient developed severe chest pain and odynophagia, 4 hours after swallowing the *N-*acetylcysteine tablet in the decubitus position on the second day in the hospital. Findings on physical examination were unremarkable. The results of electrocardiography (ECG) were normal. Upper gastrointestinal endoscopy revealed four discrete areas of ulcerations measuring approximately 1 cm at the midesophageal level. The distance between the foci and the incisors was approximately 24 cm (Figure 1).

**Figure 2:**
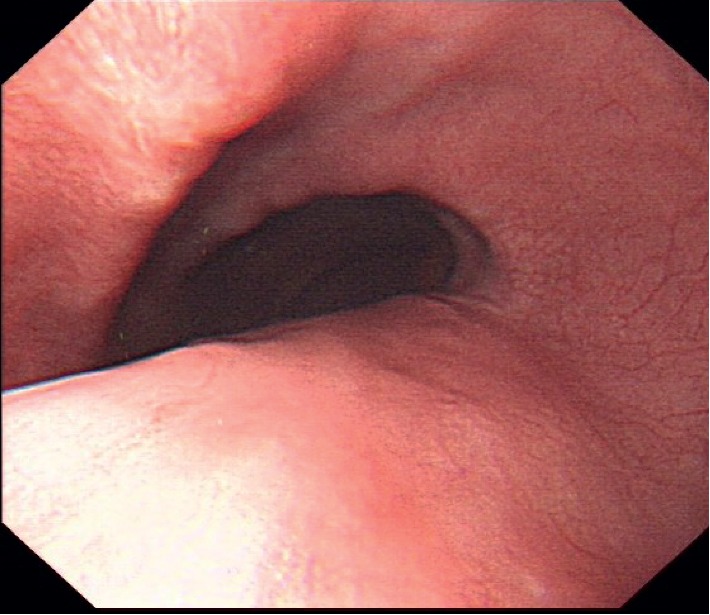
Endoscopic appearance, with healed ulcers, 20 days later.

Because of concurrent illness, the patient needed to continue taking *N-*acetylcysteine, which was administered in particle form dissolved with more water in the upright position. Meanwhile, the patient was administered pantoprazole and hydrotalcite for treatment of the ulcers. After the course of treatment, the symptoms subsided, and a follow-up endoscopy after 20 days showed that the ulcers have healed (Figure 2).

## 3. Discussion

The diagnosis of pill-induced esophagitis is based on both clinical symptoms and endoscopic findings, with the most common symptoms being acute-onset retrosternal pain or heartburn, odynophagia, and dysphagia. Endoscopy typically reveals midesophageal ulcers [3]. Delay in diagnosis may result in severe complications including gastrointestinal bleeding, stenosis, and perforation. A tablet of *N-*acetylcysteine exhibits an enteric coating, and the drug ingredients in the coating exhibit a low pH. The pKa of the carboxylic acid moiety of acetylcysteine is ∼3. The tablet may adhere to the esophageal mucosa with an insufficient amount of water in the decubitus position. After the coating is dissolved by the mucus in the esophagus, its free sulfhydryl group breaks disulfide bonds and lowers the viscosity of the mucus. Then, due to prolonged contact of the acidic drug with the esophageal mucosa, mucosa injury occurs consequently [1]. Therefore, patients who ingest the pill without a sufficient amount of water and then remain recumbent are at the highest risk of developing pill-induced esophagitis [4]. Moreover, pills tend to lodge at areas of esophageal narrowing, such as the levels of the aortic arch, the left main bronchus, and the gastroesophageal junction [5]. Most cases of pill-induced esophagitis are self-limiting and heal without intervention over 3–10 days [6].

This patient was at a marked risk of developing esophageal dysmotility due to his advanced age; moreover, he remained supine after ingesting the tablet with an insufficient amount of water. Furthermore, the shape of the *N-*acetylcysteine tablet is relatively large, which might result in a greater chance to be detained in the narrow segment of the esophagus. The lesions revealed by endoscopy were typical of pill-induced esophagitis. Although reports of pill-induced esophagitis for *N-*acetylcysteine are rare, the findings were consistent with pill-induced esophagitis and implicated *N-*acetylcysteine as the causative agent.

The present case highlights the importance of enhancing clinician awareness of drug-associated esophageal injury in patients with retrosternal pain. Moreover, patients should be educated by physicians about possible side effects of the drug, and patients should drink at least 100 mL of water after swallowing the medication while in the upright position.
